# Interventions impacting the accessibility of sexual reproductive health services for head porters in sub-Saharan Africa- A scoping review protocol

**DOI:** 10.1371/journal.pone.0289564

**Published:** 2023-08-18

**Authors:** Kimberly Jarvis, Solina Richter, Samuel Adjorlolo, Michelle Swab, Eric Tenkorang, Yuping Mao, Laura A. Chubb, Charles Ampong Adjei, William Midodzi, Adom Manu, Kwasi Torpey, Cara Spence, Pammla Petrucka

**Affiliations:** 1 Faculty of Nursing, Memorial University of Newfoundland, Newfoundland, Canada; 2 College of Nursing, University of Saskatchewan, Saskatoon, Canada; 3 Research Fellow Department of Health Studies, University of South Africa, Pretoria, South Africa; 4 Department of Mental Health, University of Ghana, Accra, Ghana; 5 Faculty of Medicine, Memorial University of Newfoundland, Newfoundland, Canada; 6 Department of Sociology, Memorial University of Newfoundland, Newfoundland, Canada; 7 Department of Communication Studies, California State University, Long Beach, California, United States of America; 8 Faculty of Education and Social Work, University of Auckland, Auckland, New Zealand; 9 Department of Public Health Nursing, University of Ghana, Accra, Ghana; 10 Department of Population, Family and Reproductive Health at the School of Public Health, University of Ghana, Accra, Ghana; 11 Department of Medicine, University of Saskatchewan, Saskatoon, Canada; World Health Organization, SOUTH SUDAN

## Abstract

Head porters working in markets in sub-Saharan Africa (SSA) are one of the world’s most vulnerable and socioeconomically disadvantaged groups. They consist predominantly of uneducated women and girls seeking to escape poverty, early marriage, and other issues of domestic violence. Most female head porters are in their reproductive years and often lack access to sexual reproductive health services (SRHS) despite being at high risk for sexually transmitted infections (STIs), unplanned pregnancies, and gender-based violence. The low priority for women and girls’ SRH in many SSA countries highlights the need to explore the factors influencing the accessibility of services for failure to do so restrains human development. An initial search of the literature was conducted and revealed no current scoping or systematic reviews on the accessibility to SRHS for female head porters in SSA. We outline a scoping review protocol, using the Joanna Briggs Institute methodology, to determine the interventions that influence the accessibility of SRHS for female head porters in SSA. The protocol is registered with Open Science Framework (https://osf.io/hjfkd). Findings will not only be valuable for female head porters but for all vulnerable female groups in SSA who experience high SRH risks and social disparities.

## Introduction

Head porters working in markets in sub-Saharan Africa (SSA) are one of the most vulnerable groups. They consist predominantly of women who have migrated from the impoverished regions in SSA to more affluent urban areas, seeking to escape poverty, early marriage, and issues of gender-based violence [1, 2]. Head porters carry loads on their heads and have locally designated/acquired names unique to the various countries in SSA. In Ghana, for example, head porters are called ‘kayayei’; while in Nigeria, they are referred to as ‘alabaru’ [1, 3]. Male porterage also exists but they mainly push ‘pulling trucks’ and generally do not carry loads on their heads. The number of head porters in any geographical area is often unknown, as the ‘trade’ is not regulated in SSA. In 2017, it was estimated that 160,000 head porters resided in Accra, Ghana with 15,000 more young women predicted to enter the occupation yearly [4].

Head porterage is a strategy for economic survival [5, 6]. Head porters work in markets, delivery stations, and/or car parks where their services are needed, carrying goods, and belongings for a fee [7]. Typically, they carry more than their own weight on their heads from dusk to dawn, up and down the maze of what constitutions the market area. Working as a head porter is considered profitable, which encourages women to migrate to ‘richer’ urban centers to earn a meager living [8]. Due to their disadvantaged socioeconomic background, head porters often feel obligated to meet the financial needs of their kin and subsequently remit portions of their earnings to support their families. Additionally, some supplement their daily earnings by engaging in nocturnal sex work when the ‘normal day’s work’ is over. This survival strategy places them at risk for sexually transmitted infections (STIs), hygiene-related illnesses, gender-based and sexual violence such as rape, unplanned pregnancies, illegal abortions, and human trafficking [9].

Most head porters are uneducated, unskilled migrant women in their reproductive ages, between 15 and 49 years, who often lack access to SRHS [7, 8]. This neglected service affects their physical, psychological, sexual, and socioeconomic health and wellbeing. Evidence suggests that women commonly experience exploitation at the hands of their employers, as well as, landlords, police, commercial vehicle drivers (taxi and delivery trucks), and city guards that are employed by the local government to regulate and enforce rules in the markets [10]. Power inequities undeniably exist when female head porters depend on these individuals, who are often men, for their survival and wellbeing.

The literature suggests that head porters’ knowledge of SRH are low, and their access to and utilization of health services are poor [10–12]. While constraints to basic and fundamental SRHS are often rooted in insufficient financial and systematic resources, such as infrastructure and a well-functioning health system, other reasons exist that impacts access [13–15]. Access to SRHS is often limited, for example, by cultural inhibitions, perceived negative consequences arising from the use of contraceptives, especially injectable and intrauterine devices [16], fear and embarrassment of being seen at a health facility when such services are socially unacceptable as well as negative attitudes of health care providers [17].

The lack of access to SRHS is compounded by recurring epidemics and/or pandemics. During public health emergencies, human and financial resources are diverted from various health programs to respond to the crisis. This sudden diversion of resources negatively affects the delivery of SRHS as demonstrated during the recent coronavirus (COVID-19) pandemic [17, 18]. Since the pandemic, there has been decreased access to basic SRHS, and increased violations of SRH rights, while the risk for gender-based and sexual violence has significantly increased by an estimated 30% in some SSA countries [17]. It is important to consider this while acknowledging the low priority for women and girls’ SRH in many SSA countries. Historically, many SSA countries are patriarchal societies where women experience SRH inequality and inadequate access to SRHS [18].

The profound and measurable benefits of attending to women and girls’ SRH are linked to “gender equality and women’s wellbeing, their impact on maternal, newborn, child, and adolescent health, and their roles in shaping future economic development and environmental sustainability” [19, p.2642]. It is further argued that individuals have the right to make decisions related to their bodies that are “free of stigma, discrimination, and coercion. These decisions include those related to sexuality, reproduction, and the use of SRHS” [19, p.2642]. According to the United Nations 2030 Sustainable Development Goals (SDGs), accessibility to SRHS is essential for sustainable development [20]. Specifically, SDG 3; good health and wellbeing, targets 3.7 and 3.8 are important because they ensure “universal access to SRH care services” [20, goal 3 targets. para, 11] and “universal health coverage …for all” [20, goal 3 targets. para, 12]. A 2018 report in the Lancet recommended that countries should include SRH as an essential service within the universal health coverage palette with special attention to the poorest and most vulnerable [21]. The World Health Organization (WHO) is striving

for a world where all women’s and men’s rights to enjoy sexual and reproductive health are promoted and protected, and all women and men, including adolescents and those who are underserved or marginalized, have access to sexual and reproductive health information and services

[22, para.2].

In many SSA countries, migrant female head porters may be displaced and have unsafe housing, and working conditions as their employers frequently have power over them, inhibiting their access to needed resources. Additionally, as previously mentioned, these women also experience gender-based violence, unplanned pregnancies, and STIs [9]. This places head porters in what some may call a ‘precarious environment’ [2]. Improving SRH access and outcomes (particularly to contraception and women’s empowerment) is essential to achieving population health.

The Minimum Initial Service Package (MISP), is an approach designed by a global coalition, the Inter-Agency Working Group for Reproductive Health in Crisis, to advance comprehensive SRH and SRH rights in precarious environments [23]. This review will use this approach as a guide for extracting data since female migrant head porters are often in unstable environments which threaten access to SRHS. The MISP objectives include:

- “Ensure the health sector identifies an organization to lead the implementation of the MISP,
- Prevent sexual violence and respond to the needs of survivors,
- Prevent the transmission of and reduce morbidity and mortality due to HIV and other STIs,
- Prevent excess maternal and newborn morbidity and mortality,
- Prevent unintended pregnancies,
- Plan for comprehensive SRH services, integrated into primary health care” [23, para. 4].

Despite limited interventions specifically targeting head porters [24, 25] there are numerous efforts directed towards vulnerable women and girls with unmet SRH needs in SSA.

Educational, community-based, primary prevention, empowerment-based, psychological/counseling, income generating training programs, and development of SRH risk-reduction messages are the SRH interventions found in the literature [26–32]. Additionally, the use of medicine shops/vendors as an alternative for accessing family planning methods was advanced as an intervention to increase access to SRHS [33, 34]. The identification of gaps and knowledge of what SRHS are available and why current interventions succeed or fail, can set the foundation for improved SRH outcomes and access to services. An initial search found no current scoping or systematic reviews in PROSPERO, MEDLINE, the Cochrane Database of Systematic Reviews, or the Joanna Briggs Institute (JBI) Evidence Synthesis. There is an urgent need to conduct a scoping review on the SRH service interventions’ accessibility to female head porters in SSA because the health of women and girls is relevant to promoting human development (i.e., gender equality, agency, good health for all, etc.,) [35]. The findings of this review would be valuable to not only head porters, but all vulnerable women groups in SSA needing to access SRHS since the root cause of inadequate access to SRHS is often linked to gendered issues, power imbalances, and social disparities.

## Material and methods

The Joanna Briggs Institute methodology for scoping reviews [36] will be utilized in this review. The protocol is registered with Open Science Framework (https://osf.io/hjfkd).

### Review question(s)

What interventions impact the accessibility of SRHS for head porters in sub-Saharan Africa?

### Inclusion and exclusion criteria

The following is a described list of inclusion criteria. All other articles will be excluded from this review.

#### Participants

This review will be taken into consideration papers that include female head porters who work in SSA markets carrying goods and wares on their heads from one location to another for a fee. While traditionally both males and females participate in head porterage, only females will be considered since they comprise most of the workforce and have SRH needs that differ from their male counterparts.

#### Concept

Accessibility of SRH interventions for head porters is the concept being examined in this scoping review. For the purpose of this review, we define access as: 1. “affordability (direct and indirect cost of health), 2. physical accessibility (e.g., opening hours, geographical placement/reach), 3. acceptability (e.g., willingness to seek services), and 4. social or cultural factors (e.g., language or the age, sex, ethnicity, or religion of the health provider)” [37, para.4].

The types of interventions considered in this review are primary (health promoting, preventing disease or injury), secondary (early detection of a disease or injury or preventing it from becoming worst), and tertiary (managing a disease or injury) SRH interventions. We further adopted the 2021 United Nations Population Fund (UNFPA) definition for good SRH as:

a state of complete physical, mental, and social well-being in all matters relating to the reproductive system. It implies that people are able to have a satisfying and safe sex life, the capability to reproduce, and the freedom to decide if, when, and how often to do so. To maintain one’s sexual and reproductive health, people need access to accurate information and the safe, effective, affordable, and acceptable contraception method of their choice. They must be informed and empowered to protect themselves from sexually transmitted infections. And when they decide to have children, women must have access to services that can help them have a fit pregnancy, safe delivery, and healthy baby. Every individual has the right to make their not own choices about their sexual and reproductive health

[38, para.1].

#### Context

This review will consider papers that discuss the impact of head porterage on females who work in urban and rural African SSA markets. A total of 42 SSA countries and six island nations will be included [39, 40].

#### Types of sources

Quantitative, qualitative, and mixed method studies, program evaluations, quality improvement reports, dissertations, theses, peer reviewed conference papers, and opinion pieces will be considered for inclusion.

Unpublished reports will be incorporated in this review but will be limited to national and international governmental and non-governmental websites, which work with women in SSA around SRHS, such as the WHO and the United Nations Population Fund (UNFPA).

Acknowledging the substantial attention to head porterage in SSA since the mid-1980s and the advocacy for women and girl’s SRH rights and interventions during the era targeting the United Nations 2000 Millennium Development Goals [41], studies published between 2000 to 2023 will be included. French, English, and Portuguese articles will be included as many SSA countries speak these languages, in addition to their mother tongue.

#### Search strategy

The search for evidence will be conducted by the library scientist on the review team, in consultation with other team members. The aim of the search strategy is to find both published and unpublished studies. The search will include a variety of subject databases in the medical and social sciences fields, such as:

- Ovid Medline
- Embase (via embase.com)
- CINAHL Plus (via EBSCOhost)
- APA PsycInfo (via EBSCOhost)
- Anthropology Plus (via EBSCOhost)
- ERIC (via EBSCOhost)
- SocINDEX (via EBSCOhost)
- Women’s Studies International (via EBSCOhost)
- International bibliography of the social sciences (via ProQuest)

Keywords and controlled vocabulary search terms were selected in consultation with a subject expert and were revised based on preliminary search results. Given the results of the draft search strategy in Ovid Medline, it is anticipated that many articles and reports relating to this topic will not be indexed in the subject databases. Therefore, extensive Google Scholar searches will be conducted to capture this literature. Google Scholar is the most inclusive and comprehensive of the major academic literature indexes. Forward and backward citation tracing will also be of crucial importance and will be conducted for all included studies. Backward citation tracing involves a review of reference lists of all included studies. Forward citation tracing looks at citing articles and will again be conducted in Google Scholar. The search for grey literature and unindexed studies will include the following websites and platforms:

- ProQuest Dissertations and Theses
- OATD: Open Access Theses and Dissertations
- Bielefeld Academic Search Engine
- OAIster
- OpenGrey
- African Journals Online
- African Index Medicus (AIM)
- ELDIS

Draft searches can be found in supporting information S1 File: Search strategy in Ovid Medline and S2 File: Search strategy of preliminary grey literature. Following the search, citations will be uploaded into Endnote X8, and duplicates removed. The remaining citations will be uploaded to Covidence. Titles and abstracts will be checked against the inclusion criteria by two independent reviewers. To ensure agreement among the reviewers, the first 50 titles and abstracts will be pilot tested. Only relevant papers will be reviewed in full text by two independent reviewers and the reasons for exclusion will be noted and recorded in the final report. Disagreements that arise at any stage of the screening process will be addressed by way of a third reviewer or through discussion. Disagreements will provide the chance to reinforce the screening process and inclusion criteria. The PRISMA Extension for Scoping Reviews (PRISMA-ScR) [42] flow diagram will be used to display the search results in the final report.

#### Data extraction

At the start of data extraction, all reviewers will review and extract data for the first 10 papers to ensure consistency and clarity between reviewers using the data extraction tool developed in supporting information S3 File: Data extraction instrument. Once data extraction issues are resolved, data will be extracted from all included papers by two independent reviewers in accordance with JBI methodology. The following data will be extracted, as applicable: author(s), year of publication, the purpose of the study, study design, target population characteristics /sample size, study setting/location, study intervention details as well as gaps, key findings, and study recommendations.

A third reviewer will decide on any disagreements. Authors, of included articles, will be contacted when deemed necessary (i.e., missing data). All necessary revisions throughout the data extraction process will be noted in the scoping review report.

#### Data analysis and presentation

The extracted data will be displayed in a table, supplemented with a descriptive summary, aligning with the objectives of this scoping review. Results will provide the foundational knowledge for future interventions addressing the accessibility of SRHS for head porters in SSA.

## Supporting information

S1 File Search strategy in Ovid Medline. (DOCX)

S2 File Search strategy of preliminary grey literature. (DOCX)

S3 File Data extraction instrument. (DOCX)

S1 Checklist Preferred Reporting Items for Systematic reviews and Meta-Analyses extension for Scoping Reviews (PRISMA-ScR) checklist. (PDF)
