# Supplementary material for: Interventions impacting the accessibility of sexual reproductive health services for head porters in sub-Saharan Africa- A scoping review protocol
Source: PLoS One. 2023 Aug 18;18(8):e0289564. doi: 10.1371/journal.pone.0289564 (PMC10437979; doi:10.1371/journal.pone.0289564)
Supplement: S1 File — (DOCX) [file pone.0289564.s001.docx]

**S1: Search strategy in Ovid Medline**

Database: Ovid MEDLINE(R) and Epub Ahead of Print, In-Process, In-Data-Review & Other Non-Indexed Citations and Daily <1946 to November 23, 2022>

Search Strategy:

--------------------------------------------------------------------------------

1 (kayayei or 'kaya yei' or kayayoo or 'kaya yoo').ti,ab. (5)

2 alabaru.ti,ab. (0)

3 head porter*.ti,ab. (11)

4 human porter*.ti,ab. (1)

5 female porter*.ti,ab. (0)

6 women porter*.ti,ab. (0)

7 market porter*.ti,ab. (1)

8 human bread basket?.ti,ab. (0)

9 headload*.ti,ab. (3)

10 head load*.ti,ab. (86)

11 pedestrian load*.ti,ab. (4)

12 ('market work*' and (subsaharan or 'sub saharan' or angola or benin or botswana or 'burkina faso' or burundi or 'cabo verde' or cameroon or 'central african republic' or chad or comoros or congo or 'cote d'ivoire' or eritrea or eswatini or swaziland or ethiopia or gabon or gambia or ghana or accra or kumasi or guinea or kenya or lesotho or liberia or madagascar or malawi or mali or mauritania or mauritius or mozambique or namibia or niger or nigeria or lagos or kano or ibadan or rwanda or 'sao tome' or senegal or seychelles or 'sierra leone' or somalia or 'south africa' or sudan or tanzania or togo or uganda or zambia or zimbabwe)).ti,ab. (9)

13 ('market employ*' and (subsaharan or 'sub saharan' or angola or benin or botswana or 'burkina faso' or burundi or 'cabo verde' or cameroon or 'central african republic' or chad or comoros or congo or 'cote d'ivoire' or eritrea or eswatini or swaziland or ethiopia or gabon or gambia or ghana or accra or kumasi or guinea or kenya or lesotho or liberia or madagascar or malawi or mali or mauritania or mauritius or mozambique or namibia or niger or nigeria or lagos or kano or ibadan or rwanda or 'sao tome' or senegal or seychelles or 'sierra leone' or somalia or 'south africa' or sudan or tanzania or togo or uganda or zambia or zimbabwe)).ti,ab. (2)

14 ('market labor*' and (subsaharan or 'sub saharan' or angola or benin or botswana or 'burkina faso' or burundi or 'cabo verde' or cameroon or 'central african republic' or chad or comoros or congo or 'cote d'ivoire' or eritrea or eswatini or swaziland or ethiopia or gabon or gambia or ghana or accra or kumasi or guinea or kenya or lesotho or liberia or madagascar or malawi or mali or mauritania or mauritius or mozambique or namibia or niger or nigeria or lagos or kano or ibadan or rwanda or 'sao tome' or senegal or seychelles or 'sierra leone' or somalia or 'south africa' or sudan or tanzania or togo or uganda or zambia or zimbabwe)).ti,ab. (2)

15 ('market labour*' and (subsaharan or 'sub saharan' or angola or benin or botswana or 'burkina faso' or burundi or 'cabo verde' or cameroon or 'central african republic' or chad or comoros or congo or 'cote d'ivoire' or eritrea or eswatini or swaziland or ethiopia or gabon or gambia or ghana or accra or kumasi or guinea or kenya or lesotho or liberia or madagascar or malawi or mali or mauritania or mauritius or mozambique or namibia or niger or nigeria or lagos or kano or ibadan or rwanda or 'sao tome' or senegal or seychelles or 'sierra leone' or somalia or 'south africa' or sudan or tanzania or togo or uganda or zambia or zimbabwe)).ti,ab. (0)

16 exp "Africa South of the Sahara"/ and ('market work*' or 'market employ*' or 'market labor*' or 'market labour*').ti,ab. (9)

17 or/1-16 (118)

18 exp Women's Health/ (31425)

19 Sexual Health/ (2253)

20 (health adj2 (women* or reproductive or sexual or female or maternal)).ti,ab. (66991)

21 exp Maternal Health Services/ (56633)

22 ((maternal or prenatal or 'pre natal or antenatal or 'ante natal' or perinatal or 'peri natal' or postnatal or 'post natal') adj2 care).ti,ab. (33541)

23 Breast Feeding/ (42795)

24 (breastfeed* or 'breast feed*').ti,ab. (45236)

25 exp Reproductive Health Services/ (43437)

26 exp Contraception/ (29051)

27 exp Contraceptive Agents/ (78180)

28 (contraception or contraceptive? or 'family planning' or 'birth control').ti,ab. (88494)

29 exp Abortion, Induced/ (42241)

30 abortion.ti,ab. (54584)

31 exp Sexually Transmitted Diseases/ (372567)

32 sexually transmitted infection?.ti,ab. (16358)

33 sexually transmitted disease?.ti,ab. (16740)

34 (hiv or 'human immunodeficiency virus').ti,ab. (362155)

35 exp "female urogenital diseases and pregnancy complications"/ (1682353)

36 (fistual adj2 (obstetric* or vaginal or rectovaginal)).ti,ab. (2)

37 Circumcision, Female/ (1633)

38 ('female circumcision' or 'female genital cutting' or 'female genital mutilation').ti,ab. (1914)

39 sexuality/ or safe sex/ or unsafe sex/ (15878)

40 (sexual* adj3 (counsel* or educat* or teach* or learn*)).ti,ab. (5458)

41 'sexual consent'.ti,ab. (149)

42 exp Sex Offenses/ (26927)

43 (rape or 'sexual violence').ti,ab. (12524)

44 or/18-43 (2473850)

45 (english or french).lg. (30876415)

46 17 and 44 and 45 (7)
