# Supplementary material for: Interventions impacting the accessibility of sexual reproductive health services for head porters in sub-Saharan Africa- A scoping review protocol
Source: PLoS One. 2023 Aug 18;18(8):e0289564. doi: 10.1371/journal.pone.0289564 (PMC10437979; doi:10.1371/journal.pone.0289564)
Supplement: S2 File — (DOCX) [file pone.0289564.s002.docx]

**S2: Search strategy of preliminary grey literature**

Google Scholar

| Search Strategy | Total results | Potentially relevant results |
| --- | --- | --- |
| allintitle: kayayei OR "kaya yei" OR kayayoo OR "kaya yoo" OR "head porters" OR "human porters" OR "female porters" OR "women porters" OR "market workers" | 249 | 17 |

ProQuest Dissertations and Theses

| Search Strategy | Total results | Potentially relevant results |
| --- | --- | --- |
| noft(kayayei OR "kaya yei" OR kayayoo OR "kaya yoo" OR "head porters" OR "human porters" OR "female porters" OR "women porters" OR "market workers") | 32 | 2 |

OATD: Open Access Theses and Dissertations

| Search Strategies (as single terms) | Total results | Potentially relevant results |
| --- | --- | --- |
| kayayei  "kaya yei"  kayayoo  "kaya yoo"  "head porters"  "human porters"  "female porters"  "women porters"  "market workers" | 30 | 6 |

Bielefeld Academic Search Engine

| Search Strategy | Total results | Potentially relevant results |
| --- | --- | --- |
| Entire Document: kayayei OR "kaya yei" OR kayayoo OR "kaya yoo" OR "head porters" OR "human porters" OR "female porters" OR "women porters" OR "market workers" doctype:(11* 13 14 15 18* 19) | 120 | 7 |

OAIster

| Search Strategy | Total results | Potentially relevant results |
| --- | --- | --- |
| kw:(kayayei OR "kaya yei" OR kayayoo OR "kaya yoo" OR "head porters" OR "human porters" OR "female porters" OR "women porters" OR "market workers") | 146 | 5 |

African Journals Online

| Search Strategy | Total results | Potentially relevant results |
| --- | --- | --- |
| kayayei OR "kaya yei" OR kayayoo OR "kaya yoo" OR "head porters" OR "human porters" OR "female porters" OR "women porters" OR "market workers" | 44 | 5 |

African Index Medicus (AIM)

| Search Strategy | Total results | Potentially relevant results |
| --- | --- | --- |
| kayayei OR "kaya yei" OR kayayoo OR "kaya yoo" OR "head porters" OR "human porters" OR "female porters" OR "women porters" OR "market workers" | 8 | 1 |

ELDIS

| Search Strategy | Total results | Potentially relevant results |
| --- | --- | --- |
| kayayei OR "kaya yei" OR kayayoo OR "kaya yoo" OR "head porters" OR "human porters" OR "female porters" OR "women porters" OR "market workers" | 161 | 1 |
