# Supplementary material for: Interventions impacting the accessibility of sexual reproductive health services for head porters in sub-Saharan Africa- A scoping review protocol
Source: PLoS One. 2023 Aug 18;18(8):e0289564. doi: 10.1371/journal.pone.0289564 (PMC10437979; doi:10.1371/journal.pone.0289564)
Supplement: S3 File — (DOCX) [file pone.0289564.s003.docx]

**S3: Data extraction instrument**

This tool will be refined and revised as needed during the data extraction process.

| Criteria | Study title |
| --- | --- |
| Author/year: |  |
| Purpose of the study: |  |
| Study design: |  |
| Target population characteristics/sample size |  |
| Study setting/location: |  |
| Study results:  1. Description and type of intervention discussed (primary, secondary, or tertiary)  2. Access to SRHS intervention (affordability, physical accessibility, acceptability, social, and cultural factors)  3. Did the intervention meet any of the minimum initial service package (MISP) for SRH in fragile environments?  This is a package developed by the Inter—Agency Working Group for Reproductive Health in Crisis and supported by UNFPA  Objective criteria for MISP:   - Ensure the Health Sector identifies an organization to lead implementation of the MISP, - Prevent sexual violence and respond to the needs of survivors, - Prevent the transmission of and reduce morbidity and mortality due to HIV and other STIs, - Prevent excess maternal and newborn morbidity and mortality, - Prevent unintended pregnancies, and - Plan for comprehensive SRH services, integrated into primary health care. |  |
| Gaps and key findings to SRHS |  |
| Study recommendations |  |
| Additional comments |  |
